# Inhibition and enhancement of linear and nonlinear optical effects by conical phase front shaping for femtosecond laser material processing

**DOI:** 10.1038/s41598-020-78373-4

**Published:** 2020-12-09

**Authors:** Ehsan Alimohammadian, Erden Ertorer, Erick Mejia Uzeda, Jianzhao Li, Peter R. Herman

**Affiliations:** Department of Electrical and Computer Engineering, University of Toronto, 10 King’s College Road, Toronto, ON M5S 3G4 Canada

**Keywords:** Applied optics, Nonlinear optics, Ultrafast photonics, Nonlinear optics

## Abstract

The emergence of high-powered femtosecond lasers presents the opportunity for large volume processing inside of transparent materials, wherein a myriad of nonlinear optical and aberration effects typically convolves to distort the focused beam shape. In this paper, convex and concave conical phase fronts were imposed on femtosecond laser beams and focussed into wide-bandgap glass to generate a vortex beam with tuneable Gaussian-Bessel features offset from the focal plane. The influence of Kerr lensing, plasma defocussing, and surface aberration on the conical phase front shaping were examined over low to high pulse energy delivery and for shallow to deep processing tested to 2.5 mm focussing depth. By isolating the underlying processes, the results demonstrate how conical beams can systematically manipulate the degree of nonlinear interaction and surface aberration to facilitate a controllable inhibition or enhancement of Kerr lensing, plasma defocussing, and surface aberration effects. In this way, long and uniform filament tracks have been generated over shallow to deep focussing by harnessing surface aberration and conical beam shaping without the destabilizing Kerr lensing and plasma defocussing effects. A facile means for compressing and stretching of the focal interaction volume is presented for controlling the three-dimensional micro- and nano-structuring of transparent materials.

## Introduction

The nonlinear interactions inherent in the ultrafast laser processing of transparent materials entails a wide range of distorting effects wherein laser energy can be dissipated into a myriad of three-dimensional (3D) shapes that deviate far from the typical Gaussian-shaped focal volume. The distortions arise on multiple levels, beginning with spherical aberration of the sample surface^1^ (hereafter, surface aberration) that stretches the focal volume forward or intensity clamping^2^ that shifts the interaction backward with respect to the laser propagation direction. Nonlinear interactions such as the Kerr^3^ effect draw the focal interaction volume both forward and backward, while plasma defocussing breaks apart the interaction volume. The accumulation of material defects, incubation sites, heat, and other physio-chemical effects can further assemble novel 3D structures with multi-pulse exposure by high repetition rate pulse trains^4^. Despite a seemly unlimited number of interaction pathways, the resulting material modification can be readily manipulated to induce refractive index structures^5^, volume nano-gratings^6–8^, nano-voids^9^, elongated filaments^10^, and nano-channels^11^ in reproducible ways. Femtosecond laser processing is a key enabling technology^12^ today for micro- and nano-structuring of transparent bulk glasses including thin films^9^, and optical fibres^13^ that serve in a variety of applications from manufacturing of biomedical^14^ and photonic^15,16^ devices to scribing^17^, cutting^18^, welding, and surgical^19^ applications.

In optical microscopy, surface and other forms of aberration have been countered in a variety of practical ways^20^ that have been exploited in ultrafast laser processing to improve on the resolution of structuring the inside of transparent materials. Compensation collar lenses^21^ and spatial light modulators (SLMs)^20,22–24^ have been used for structuring diamond defects^22^, writing low-loss optical waveguides^23^, and fabricating 3D photonic crystals^24^. In contrast, spatio-temporal beam shaping seeks to influence the form of the laser interaction volume. Astigmatic beams generated by cylindrical lenses^25^, slit apertures^26^ or SLMs^27^ have been used to counter the asymmetric focal volume and form cylindrically symmetrical waveguides^27^. SLMs further enable active beam shaping during laser scanning to correct the waveguide distortion at a substrate facet^28^ or to separate the beam to enable parallel processing on multi-focal positions^29,30^. Iterative methods provide flexible means for optimizing the shape of lab-on-a-chip sub-components^31^ while accelerated beams^32^ offer 3D shaping of curved trenches. Alternatively, elongation of the interaction volume into a filament shape is attractive for forming vertical waveguides^33^, stress-cleaving arrays^17,34^, and thick welding^35^ seams while Bessel-like beams^36–39^ with high aspect ratio are useful for high speed cutting of glass^40^, or opening of long and narrow holes in glasses^11,41^, and polymers^38^.

Beam shaping of ultrashort pulsed lasers is typically applied without consideration of the multiple and competing nonlinear processes that evolve inside of transparent materials to distort the beam propagation and dissipation physics. Evidence on the influence of such beam shaping can be seen in the balance of supercontinuum generation and multiphoton-induced damage that could be switched by the numerical aperture (NA) of the focussing beam as reported by Mazur et al.^42^. Kashyap et al. applied dual-beam focussing to reduce surface aberration and temporal stretching and thereby inhibit filament formation due to Kerr lensing^43^. Kammel et al*.* manipulated the space–time beam properties to diminish self-focussing and filament formation^44^ while Vitek et al*.*^45^ and Salter et al*.*^46^ demonstrated the nonreciprocal writing or directional effect of ultrafast laser interactions by pulse front tilt. Patel et al*.* further applied pulse front tilt to enable or switch off the nano-grating formation in fused silica glass^47^. In such studies, the underlying response of a single interaction pathway such as Kerr lensing, multiphoton absorption, avalanche ionization, plasma defocussing, surface aberration, material incubation, and heat-accumulation, amongst many others, are challenging to separate and follow independently at the threshold of laser material modification. Exerting control over the morphology and structural form of the laser modification volume therefore remains an open challenge where selectivity on inhibiting or enhancing the individual channels amongst the multiple, interconnected pathways is a major goal to provide consistent processing inside of a transparent substrate.

**Figure 1:**
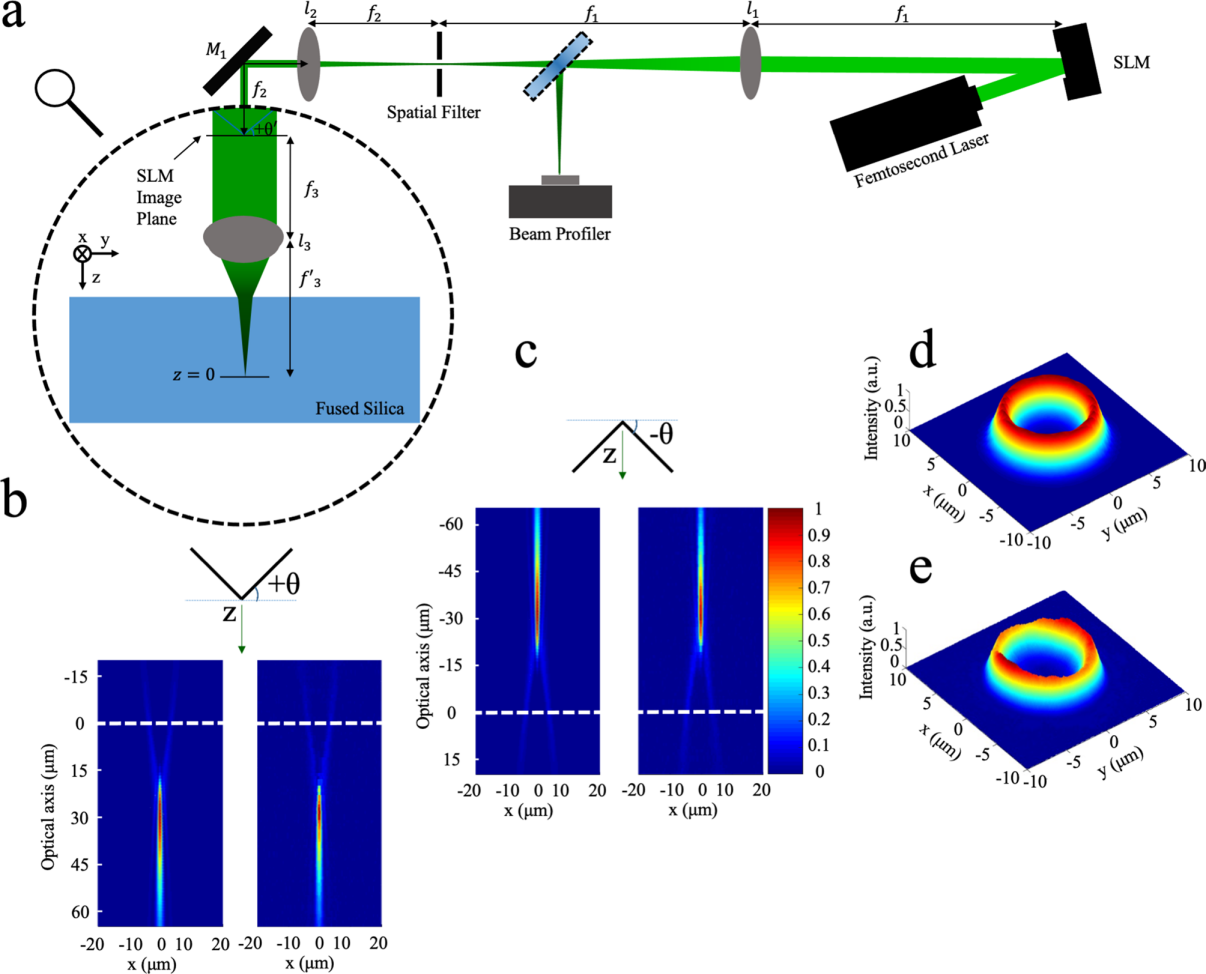
Femtosecond laser arrangement for generating and focussing Gaussian–Bessel–Vortex beams. Schematic arrangement (**a**) for conical phase front beam shaping showing a 6f lens imaging (${l}_{1}$, ${l}_{2}$, and ${l}_{3}$) to project the SLM-shaped beam to inside of a glass substrate (z = 0 plane). Simulated (left) and recorded (right) longitudinal profiles of the focussed Gaussian-Bessel beams are compared for complimentary conical phase front angles of θ =  + 0.34 mrad (**b**) and − 0.34 mrad (**c**) with laser propagation downward. Dashed line (white) marks the paraxial focal plane (z = 0) where transverse profiles of zero-angular momentum vortex beams were simulated (**d**) and recorded (**e**). Here, laser interactions are dominated by the Gaussian-Bessel beam, whose peak intensity is ~ 18$\times$ higher than the peak intensity of the Vortex beam (i.e. z = 0 position in (**b**, **c**)). Intensity profiles were recorded near the Fourier plane of lens ${l}_{1}$ with a flip mirror and translating beam profiler.

The objective of the present paper was to favourably manipulate the focal volume shape of ultrafast laser interactions as surface aberration and nonlinear optical effects played out differently over shallow to deep focussing positions inside of wide bandgap fused silica glass. SLM beam shaping was selected (Fig. 1a) to generate both positive and negative conical phase fronts and favourably distort the typical Gaussian focal spot when transformed by the fabrication lens into the Fourier domain. Vortex beams (Fig. 1d,e) were formed with Gaussian–Bessel like intensity profiles projecting either backward (Fig. 1c) or forward from (Fig. 1b) the focal plane. This approach offered a facile means of stretching the focal interaction volume for tuning laser ‘filament’ length or for countering surface aberration effects. The internal morphology resulting from the shaped beams was examined for single pulse exposure at focal depths tested up to 2.5 mm. The positive and negative axial stretching could be systematically tuned to counter or enhance surface aberration and Kerr-lensing effects, and thereby separate the interplay between these competing effects towards creating long and uniform filament tracks or highly confined focal volume, tailored for any focal depth.

## Results

The Gaussian beam profile of femtosecond laser pulses was modified (see Fig. 1a) by a phase-only SLM and beam delivery system to structure the internal volume of fused silica substrates (see “Methods”) under single-pulse exposure. To uncover the changing role of surface aberration, Kerr lensing and plasma defocussing effects, the influence of positive (Fig. 1b) and negative (Fig. 1c) conical phase fronts on the interaction volume was examined over widely varying focal depth, generating vortex beams of zero-angular momentum (Fig. 1d,e). However, the observed and simulated intensity profiles shown in Fig. 1b,c for complementary conical angles of θ = + 0.34 and − 0.34 mrad, respectively, the highest intensity to appear in the Bessel-like filament shapes positioned either before (Fig. 1c) or after (Fig. 1b) the vertex beam position, respectively. Such small conical angle thus offered an eightfold stretching of the otherwise Gaussian beam shape from 2.6 µm depth of focus (DOF) to ~ 22 µm length.

### Morphology induced by focussed Gaussian beam

**Figure 2:**
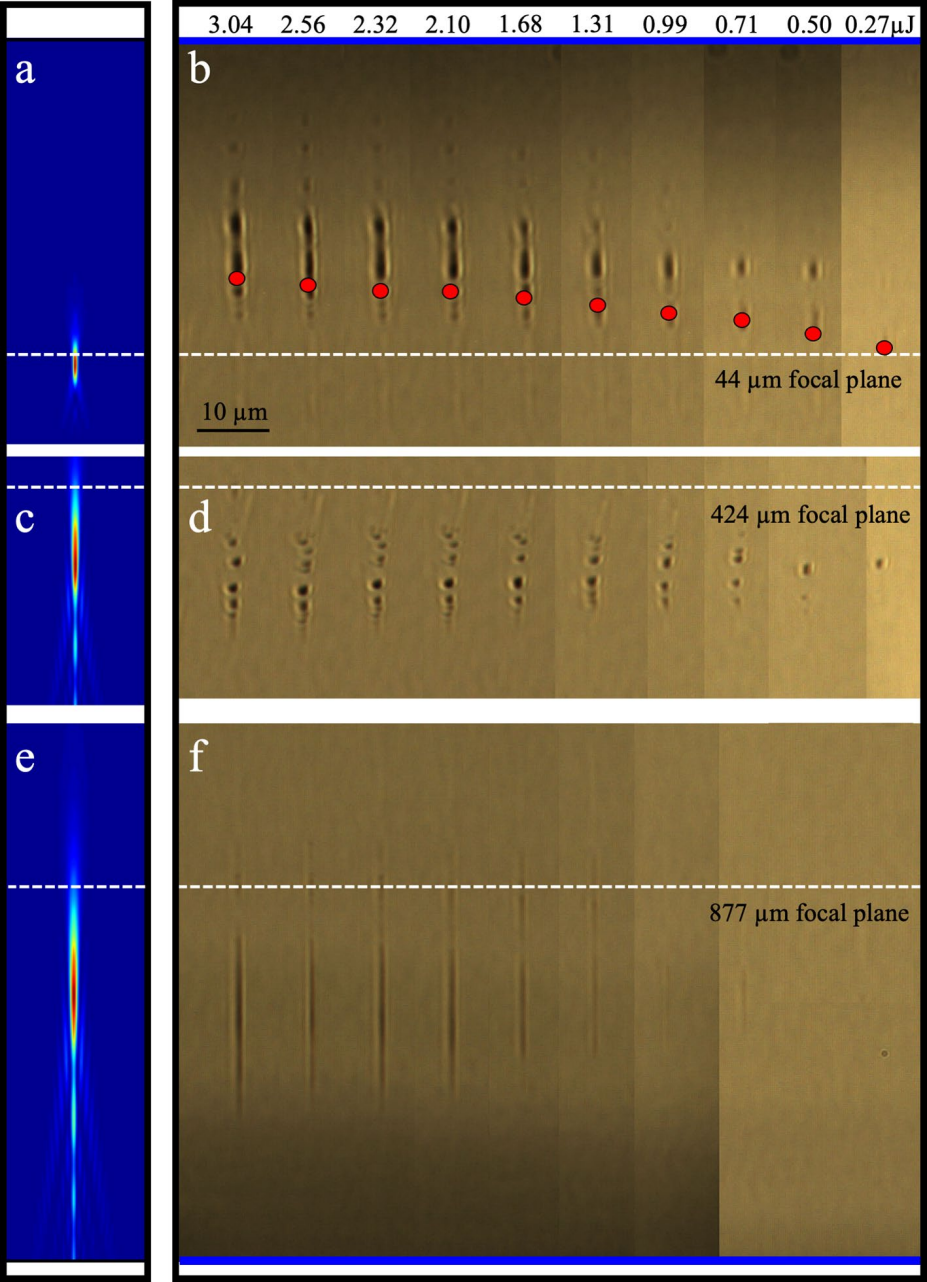
Laser modification of fused silica as distorted by Kerr lensing, plasma defocussing and surface aberration. Simulated longitudinal profile of focussed Gaussian beam under surface aberration for focussing depths of 44 µm (**a**), 424 µm (**c**), and 877 µm (**e**), and the corresponding optical microscope images (**b**, **d**, and **f**) at similar respective depths, of laser modification tracks observed under backlighting. Interaction of single laser pulses of varying energy (0.27 to 3.04 µJ) were distorted by Kerr self-focussing (**b**), plasma defocussing (**d**) and surface-aberration elongation (**f**) relative to the paraxial focal plane (white dashed line). The surface aberration simulation indicates stretching of the DOF from DOF(0,0) = 2.6 µm (without aberration) to ~ 4, 13, and 32 µm for the respective deeper focal depths. The top and bottom surfaces of the 1 mm thick substrate are marked by blue lines in (**b**, **f**), respectively. The scale bar of 10 µm applies to all images.

A baseline reference for the internal glass modification by a single pulse exposure is presented in Fig. 2 for the traditional Gaussian beam shape ($\uptheta$ = 0 mrad). The optical images were selected from a wide data range of varying pulse energy and focal depth to identify three classes of interaction as presented for the depths of 44 µm (Fig. 2b), 424 µm (Fig. 2d), and 877 µm (Fig. 2f). Over the data set, the modification zones grew both in contrast and length with increasing pulse energy tested up to 3.04 µJ. The diameter of the filament modification zone varied in a small range of 0.8 to 2.6 µm, corresponding well with the diffraction-limited spot size of ${w}_{0}$ = ~ 0.8 µm. The axial morphology was highly varied, extending from 2 to 37 µm in length and transitioning from uniform, long tracks under deep focussing (Fig. 2f) to segmented and highly contrasting tracks at shallow focussing depth (i.e. distance from sample surface to peak intensity of focussed beam). The simulated focal intensity profiles in Fig. 2a,c,e (see “Methods”) show the strong influence of surface aberration on stretching and shifting the focussed beam downward from the paraxial focus (white dashed line). Comparison with the laser-formed tracks unveil the dominating physics underlying the beam shaping with changing pulse energy and focal depth.

One can see the laser modification zones have closely followed the simulated intensity profile (Fig. 2e) only for the deepest focal depth (Fig. 2f). At this depth, the modification zone only increases in length and contrast with increasing pulse energy. The centre position remains locked in position as defined by the simulated intensity profile, stretched axially by surface aberration from 4.0 µm DOF at 44 µm depth (Fig. 2a) to ~ 32 µm at 877 µm depth (Fig. 2e). The spreading of intensity by aberration into long filament tracks is thus seen to inhibit Kerr and plasma focussing effects.

The bright and dark zones of modification in Fig. 2 typically indicate positive and negative changes in refractive index. For 877 μm focussing depth (Fig. 2f), the morphology resembles processing in Regime 2 as defined^48^ for the case of many overlaying pulse exposures. Our 250-fs pulse duration precludes Regime 1 processing. Moreover, the dramatic stretching of the focal volume by surface aberration lowers the local energy fluence to avoid formation of the disruptive morphology as defined by Regime 3. Hence, the zone of modification for deep focussing (Fig. 2e) can be modelled by the linear optics of aberrated beam shape wherein energy dissipation is dominated by multiphoton and avalanche ionization^49^ without inducing distortions from Kerr lensing or plasma defocussing.

In contrast to Fig. 2f, Kerr lensing and plasma defocussing are strongly evident over the same exposure range as presented at 44 μm depth (Fig. 2b). The modification at near-threshold 270 nJ exposure corresponds closely with the critical pulse energy for self-focussing (221 nJ) inferred from Ref. 10 (see “Methods”). The modification zone aligns closely with the simulated intensity profile, with shallow DOF of 4 µm (Fig. 2a) having negligible surface aberration. With increasing pulse energy, the filament tracks stretch to 24 µm in length and shift upward by as much as 24 µm from the geometric focus (white dashed line) for the largest exposure of 3.04 µJ (Fig. 2b). A simulation of the focal shift, based on the self-focussing critical power (see “Methods”) and the focus spot size^50^, is seen (Fig. 2b; solid red circles) to approximately follow the observed track positions for increasing pulse energy. Kerr lensing thus dominates in stretching and shifting the laser interaction zone for shallow focussing. The axial breakup of the filament tracks further attest to an interplay of plasma defocussing with the Kerr-lensing effect for this shallow focussing case. More advanced modelling^44,49^ of the nonlinear beam propagation and dissipation effects are thus necessary in the shallow focussing cases to predict the shape and morphology of the modification zones due to the additional Kerr and plasma focussing effects.

At intermediate focal depth of 424 µm, the surface aberration (Fig. 2c) elongated the focal zone to 13 µm, thus lowering the peak intensity exposure and weakening the expected Kerr and plasma focussing effects. The resulting filament structures (Fig. 2d) are aligned within the simulated intensity profile, without notable Kerr shifting or stretching effects. However, the structures show a reduced length and lower contrast in comparison with the 44 µm depth case (Fig. 2b) and have become segmented on ~ 2 µm spacing. Hence Kerr focussing and plasma defocussing effects have not been entirely inhibited at this focussing depth.

The focal distortion effects in Fig. 2 present a transition from Kerr stretching and upward focal shifting effects dominating at shallow focal depth (i.e. 44 µm, Fig. 2b) to downward shifting and stretching effects being controlled by surface aberration for deep focussing (i.e. 877 µm, Fig. 2f). Kerr and plasma focussing effects are thus inhibited for deep focussing when surface aberration can distribute a fixed laser pulse energy over sufficiently long focal length.

### Morphology induced by focussed Gaussian–Bessel beam

**Figure 3:**
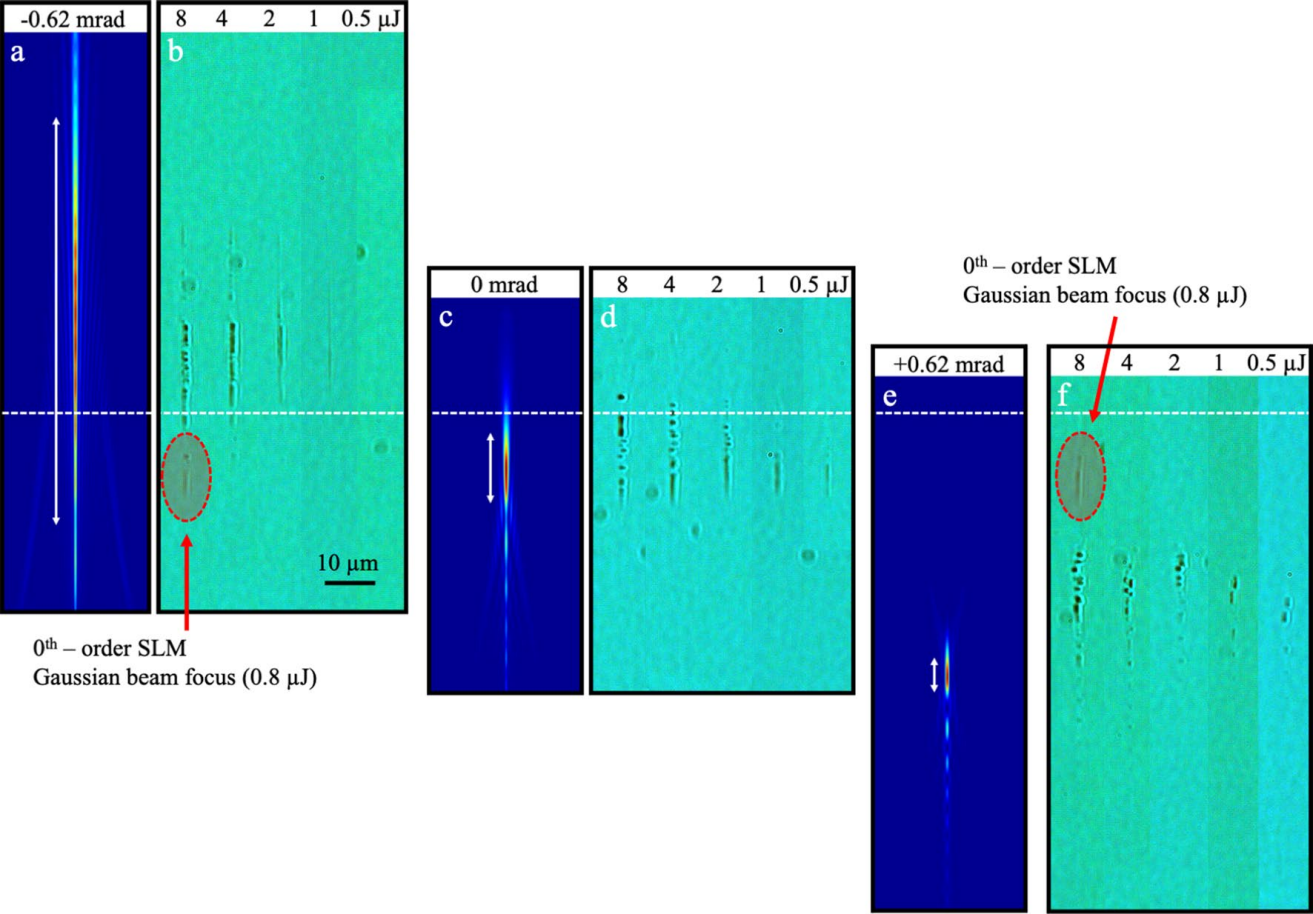
Enhancement and inhibition of surface aberration, Kerr lensing and plasma defocussing by conical phase front shaping. Longitudinal beam profiles simulated at 600 µm focussing depth in fused silica showing the combination of surface aberration and conical phase front shaping with conical angle tuned over θ = − 0.62 mrad (**a**), 0 mrad (**c**), and + 0.62 mrad (**e**), and the corresponding optical microscope images (**b**, **d**, and **f**, respectively) of the resulting laser modification tracks observed under backlighting for similar focussing depth. The evolution of morphology with single pulse energy increasing from 0.5 to 8 µJ illustrates the differing role of converging and diverging conical phase front in compensating surface aberration (**e**), inhibiting Kerr lensing (see 1 µJ in **b**), and enhancing a combination of Kerr lensing and plasma defocussing (**f**). Parallel formation of weak tracks due to surface-aberrated Gaussian beam exposure due to 10% unmodified reflection in the SLM as noted in the highest exposure cases (red dashed oval) in (**b**) and (**f**). The white arrows mark the DOF lengths of 81, 14, and 7 µm, as calculated for − 0.62, 0, and + 0.62 mrad phase front angles, respectively.

The further beam z front (i.e. Fig.1b,c) was first examined for 600 µm focal depth (Fig. 3). Without influence of conical phase front (θ = 0 mrad), the onset of Kerr and plasma focussing effects was found to have shifted to 2 µJ pulse energy (Fig. 3d) in contrast with the ~ 0.5 µJ onset noted for 44 µm depth in Fig. 2b. Below 2 µJ energy, a uniform filament-like modification track forms inside of the predicted intensity profile (Fig. 3c), stretched to 14 µm DOF by surface aberration (arrow in Fig. 3c). Above 2 μJ, upward focal shifting and segmenting of tracks arise from nonlinear Kerr and plasma interaction (Fig. 3d).

By imposing a converging conical phase front of − 0.62 mrad angle, the focussed beam was predicted to stretch ~ sixfold into a Gaussian-Bessel shape (Fig. 3a), with the peak intensity position shifting upward from − 11 µm (Fig. 3c) to + 13 µm (Fig. 3a) with respect to the geometric focus (white dashed line). The laser modification tracks (Fig. 3b) closely followed the simulated intensity envelope (Fig. 3a) up to 2 µJ exposure, marking an inhibition of the nonlinear Kerr and plasma focussing. Segmenting of filament tracks by plasma defocussing is otherwise evident for the 4 and 8 µJ pulse energy exposures.

**Figure 4:**
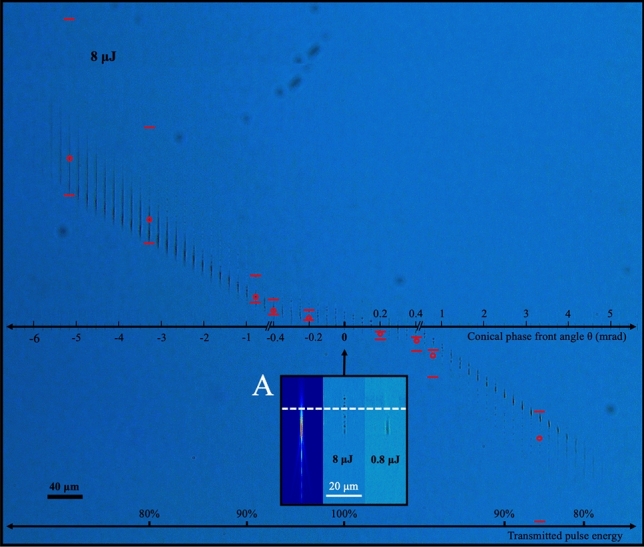
Laser modification tracks formed in fused silica by focussed Gaussian-Bessel beams with varying conical phase front angle. Sequence of modification tracks recorded under backlighting optical microscopy, generated in fused silica at 600 µm focal depth with single laser pulses of 8 µJ energy. The influence of beam shaping scales left to right as a function of conical phase front angle from − 6.6 to + 5.6 mrad. The horizontal axis marks the peak intensity position under surface aberration for the unmodified beam (θ = 0). Red circles and dashes indicate peak intensity and full width at half maximum intensity points, respectively, simulated for conical phase front angle. The bottom horizontal axis marks a reduction of the net energy transmittance through the 6f beam delivery system due to occlusion by apertures and mirrors with increasing magnitude of conical beam angle. The inset (A) compares the low (0.8 µJ) and high (8 µJ) pulse energy modification tracks with the simulated intensity profile expected under surface aberration for the unmodified Gaussian beam ($\uptheta$ = 0). The white dashed line marks the 600 µm deep, paraxial focal plane.

**Figure 5:**
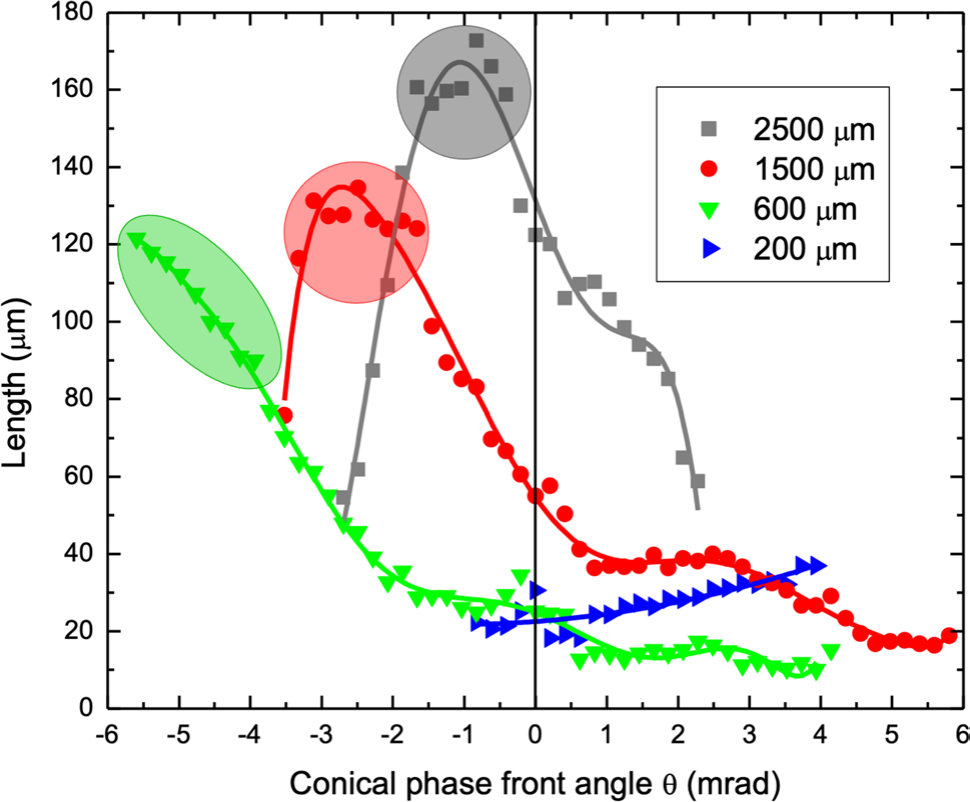
Influence of conical phase front on single-pulse filament length with increasing focal depth. Length of filament tracks formed by single pulses of 8 µJ energy, observed under varying conical phase front angles, $\uptheta$, presented for 200, 600, 1500, and 2500 µm depth. The optimal zone for long and uniform filament tracks (oval or circle sections) follows an increasingly negative conical phase front with shallower focal depth. Minimum track lengths are generated with increasingly positive conical phase front angle as depth increases. Solid lines are a guide for the eye.

**Figure 6:**
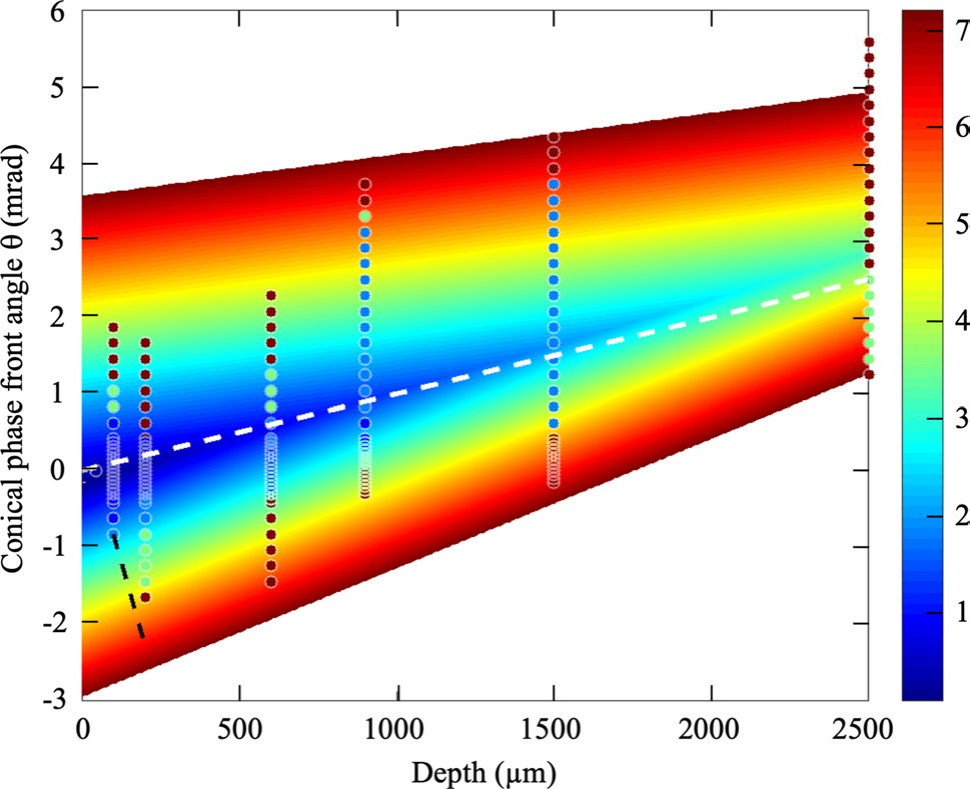
Onset laser energy for Kerr and plasma focussing under influence of surface aberration and conical phase front angle. The onset single-pulse energy (circles with false colour energy scaling) for Kerr lensing and plasma defocussing effects, tested from 270 nJ to 7.2 µJ, and plotted as a function of focal depth (44 to 2500 µm) and conical phase front angle (~ − 2.5 < $\uptheta$ < + 3.5 mrad). The onset was noted when modification tracks deviated from simulated intensity profiles. The data were represented by a model of two intersecting planes (false colour) with a vertex following closely with the conical phase front angle (white dashed line) found to compensate the surface aberration with a minimized depth of focus DOF_min_(θ_opt_(z)). The black dashed line marks the minimum (negative) conical angle observed to avoid surface ablation with increasing focal depth.

**Figure 7:**
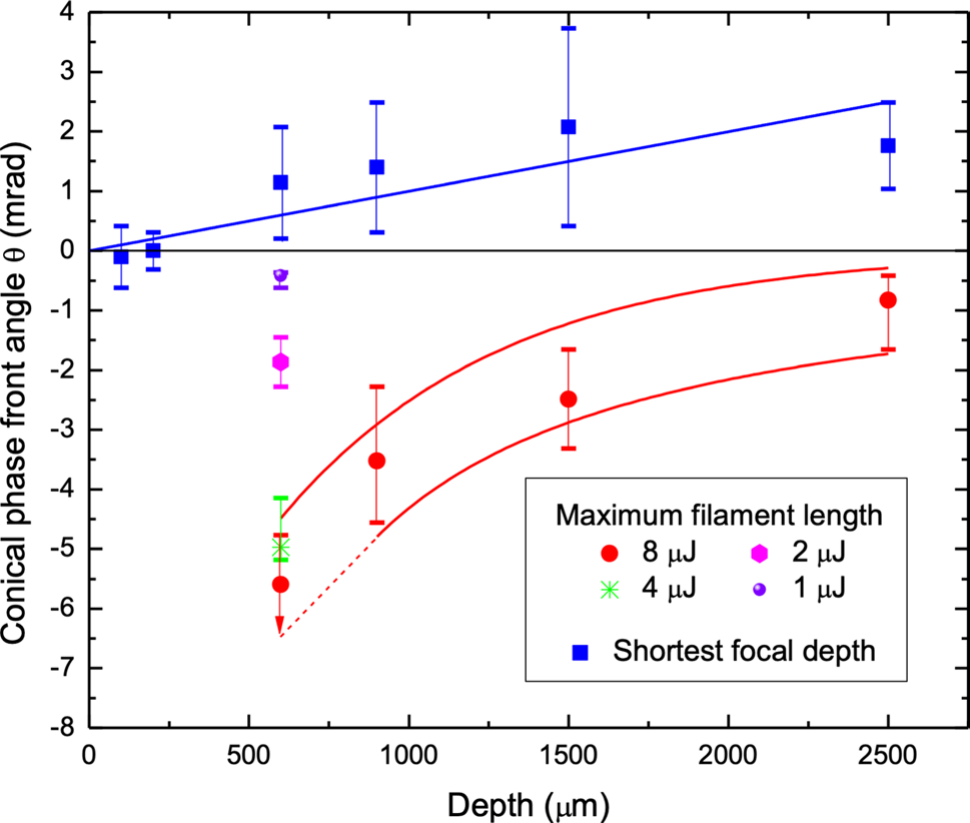
Laser processing windows for forming long uniform filaments and minimizing depth of focus, optimized by conical phase front angle. Conical phase front angle marking optimal laser processing windows for generating long and uniform filament tracks (non-square) or minimizing the track length (square) as plotted over shallow to deep focussing depth. For generating long and uniform filament tracks, an increasingly concave phase front is preferred as laser pulse energy increases from 1 to 8 µJ for 600 µm depth, and as focal depth decreases from 2500 to 600 µm. Respective vertical bars indicate angle ranges for reaching 90% of the maximum observed filament length. In contrast, convex phase fronts with monotonically increasingly conical angle yielded the shortest track length for depth increasing from 100 to 2500 µm. The data follow the solid blue line which marks the conical angle θ_opt_(z) simulated to minimize the DOF by compensating for increasing surface aberration with increasing depth (z). The blue bars represent the angle range beyond which longer tracks were discernible.

A conical phase front angle of + 0.62 mrad marked the optimal angle for compensating a significant part of the surface aberration effect at 600 μm depth. The simulated intensity profile (Fig. 3e) shows a twofold compression of the DOF from 14 µm (Fig. 3c) to 7 µm (Fig. 3e) together with a downward shift of the peak intensity position from − 11 µm (Fig. 3c) to − 52 µm (Fig. 3e) with respect to the geometric focus (white dashed line). With tighter focussing, the laser modification tracks (Fig. 3f) were observed to shift upward and break apart in contrast with the predicted intensity profile (Fig. 3e), seen over the full 0.5 to 8 µJ exposure range. The positive conical phase front thus enhances the strength of the Kerr and plasma focussing effects in contrast with the unmodified, aberrated beam (Fig. 3d). The laser modification tracks in Fig. 3 were aligned (white dashed line) by identifying the 0th-order beam reflection from the SLM. This reflection produced low-energy (0.8 μJ) modification tracks as identified (red dashed ovals) for the θ =  ± 0.62 mrad cases that aligned with the simulated intensity profile and low-energy tracks (0.5 µJ and 1.0 µJ) shown for the unmodified beam in Fig. 3d (θ = 0 mrad).

A full scaling of the conical phase front effects for θ = − 6.6 to + 5.6 mrad angle range is presented in Fig. 4 for the case of 8 μJ pulse energy and 600 μm focal depth. For reference, the beam intensity simulation (Fig. 4, inset A) for the case for unmodified beam shape (θ = 0 mrad) is reproduced from Fig. 3 together with the laser modification tracks for low (0.8 μJ) and high (8 µJ) pulse exposure. The respective cases delineate exposures well above and below the threshold for strong Kerr and plasma focussing. At 8-μJ exposure, the modification track had segmented and shifted upward by ~ 18 µm with respect to the peak of simulated intensity envelope, positioned 11 µm below the focal plane (horizontal white dashed line) due to the surface aberration. The further influence of the conical phase front (Fig. 4) showed dramatic elongation and contraction effects depending on the negative and positive sign, respectively, of the phase front angle. Leakage of 0^th^ order reflection (~ 0.8 µJ pulse energy) from the SLM provided a repeating modification pattern (magnified view Fig. 4A, right) that marked the horizontal axis for the peak intensity position at ~ 11 µm below the paraxial focus. The horizontal axis at the bottom of the figure indicates the fall-off of relative transmittance as measured through the beam delivery system with increasing magnitude of conical angle.

A Fourier simulation (see “Methods”) of the Gaussian–Bessel beam profile marked the positions (Fig. 4) of peak (red circle) and − 3 dB intensity points (red dashes) with varying conical angle. The peak intensity was shifted symmetrically from ~ 163 µm above the aberrated focal plane to ~ 152 µm below plane for the angle range from θ = − 5.6 to + 4.77 mrad, respectively. The focal elongation was also nearly symmetric, reaching 130 and 123 µm for − 3.31 and + 3.31 mrad angles, respectively. In comparison, the laser modification tracks were seen to approximately follow the predicted position of high intensity but stretching or compressing asymmetrically with increasing negative or positive phase angle, respectively.

Negative conical phase front angle (Fig. 4) provided a benefit in smoothening and stretching of the otherwise broken modification zone observed with the unmodified beam (θ = 0 mrad). The relatively uniform and long tracks were shifted downward from the predicted intensity profile (red dash and circles), possibly due to a diminishing Kerr effect that otherwise drove the 7 µm upward shift noted (Fig. 4A; 8 µJ in contrast with 0.8 µJ) for the non-modified beam shape. The combination of downward shift and non-breaking track formation thus suggests a disabling of the Kerr and plasma-defocussing effects as laser intensity falls inversely with the elongation length of the focussed Gaussian-Bessel beam (i.e. θ ≤ − 1.45 mrad).

For diverging conical phase front (positive angles), the laser interaction was shifted downward (Fig. 4), forming shorter filament tracks (i.e. 16 µm) with strong morphological contrast. As noted earlier, θ = + 0.62 mrad (Fig. 3e) marks the shortest expected DOF (7 µm) arising from conical phase front compensation of the surface aberration. The tighter focussing appears to enhance the Kerr effect by shifting the modification zone to the upper – 3 dB intensity points of the Gaussian–Bessel beam. Plasma defocussing also manifests lower in the track, seen only at low contrast (near the red open circles). Beam stretching by higher angles (i.e. θ >  + 2.9 mrad) was predicted to inhibit the Kerr lensing and plasma defocussing effects and provide long and uniform tracks. However, the simulated intensity profiles showed pronounced interference beating between the aberration and conical phase front effects for large positive conical beam angles (i.e. θ >  + 2.5 mrad) and may be responsible for breaking apart the beam, leading to observation of short filament tracks for all positive conical angles. Intensity clamping effects will also be asymmetric for the positive and negative conical angles, wherein the laser beam must pass first through the zeroth order Vortex beam at the paraxial focal plane before forming into the Gaussian–Bessel like filament in the case of positive conical angle.

The influence of conical phase front shape at lower exposures of 1, 2, and 4 µJ yielded similar morphological trends (Supplementary 1, Fig. S1) of elongation and compression on the track lengths when focussed at the same 600 μm depth. Overall filament length and structural contrast were diminished as expected with weaker pulse energy than in the 8 µJ case (Fig. 4). The onset of Kerr and plasma focussing effects were thus narrowed into smaller angular zones of phase front angle. Consequently, the optimal zone for maximizing the length of a non-broken filament scaled monotonically as: − 5.6 mrad at 8 µJ (Fig. 4), − 5 mrad at 4 µJ (Fig. S1), − 1.86 mrad at 2 µJ (Fig. S1), and − 0.41 mrad at 1 µJ (Fig. S1) pulse energy. Positive conical phase front angles in the range of + 0.26 to + 1.45 mrad provided the shortest track lengths due to the compensation of conical beam distortion against the surface aberration (i.e. θ + $\cong$ 0.62 mrad) for this 600 μm deep focussing case.

### Energy and depth compensation of filament tracks

In pursuit of generating long and uniform filaments, the laser modification zones with approximately uniform morphology were assessed from images such as Fig. 4 and Fig. S1 with track lengths plotted as a function of the conical phase front angle. Results for pulse energy scaling (1 to 8 µJ) at 600 μm depth are plotted in Fig. S2 while results for depth scaling (200 to 2500 μm) with 8 µJ pulse energy are presented in Fig. 5.

A systematic evaluation of the modification track lengths over all laser pulse energies (1 to 8 µJ) and phase front angles (− 6.6 to + 6.6 mrad) summarizes the observations for 600 μm deep focussing in terms of maximizing or compressing the DOF of laser interaction for each energy (Supplementary 2, Fig. S2). Without beam modification (θ = 0), the laser track length increases from ~ 4 to 25 µm with pulse energy rising from 1 to 8 µJ (Fig. S2). The smooth track morphology at low energy breaks into segments as Kerr and plasma focussing effects take over above 2 µJ pulse energy (Fig. 3d). These nonlinear effects were clearly inhibited by negative conical angles, enabling the longest and most uniform tracks to form. Maximum lengths of 122, 44, 22, and 12 µm (coloured ovals, Fig. S2) are reported for the respective energies of 8, 4, 2 and 1 µJ. In this range, conical phase front angles were tuned to increasing negative angles of − 5.60, − 4.97, − 1.87, and − 0.41 mrad, respectively, enabling a full harnessing of the available laser pulse energy without distortion by Kerr lensing and plasma defocussing. The slope on the 8 μJ data points to potentially longer filament lengths being possible with larger negative conical angles (θ < − 5.6 mrad), but beam occlusion in the relay optics (i.e. 29% loss at θ = − 5.6 mrad in Fig. 4) precluded such observation.

Similar experiments were repeated for depths of 200, 1500, and 2500 µm. Surface aberration was an increasingly significant factor, stretching the DOF by sixfold over the respective 200 to 2500 mm depth range. For unmodified beam shape (θ = 0 mrad) and 8 μJ pulse energy, the laser modification tracks (Fig. 5) lengthened to ~ 30, 25, 55, and 122 μm for 200, 600, 1500, and 2500 µm depths, respectively. These lengths extended far outside of the aberrated intensity zones, calculated as ~ 9, 16, 42, and 55 µm DOF, respectively.

For 200 µm depth, the tracks were highly segmented and shifted by 30 µm above the aberrated intensity peak as strong Kerr lensing and plasma defocussing dominated over the surface aberration. With increasing focal depth (600, 1500, and 2500 µm), the tracks stretched and shifted to below the focal plane, transforming with surface aberration into uniform filaments without the influence of Kerr lensing or plasma defocussing. For increasing depth, the track lengths scaled predictably with the intensity profile as demonstrated for 877 µm focal depth in Fig. 2e,f, respectively.

With only minor surface aberration (DOF =  ~ 9 µm) at 200 µm focal depth, a symmetric response of track length was expected (Fig. 5) on the sign of the conical phase front unlike the asymmetry reported for 600 µm focal depth (Fig. S2 results at 8 µJ reproduced in Fig. 5). Track lengths for 200 µm depth increased monotonically (Fig. 5, blue triangle) with increasing positive angle, without following the compression effect as seen in the 600 µm deep focussing case (Fig. 5, green triangle). The positive phase front angles thus generated Gaussian-Bessel beams, but with short lengths that caused the beam to break into multiple filament tracks on the strong nonlinear processes. The influence of negative conical angles was obscured by surface ablation beginning at θ = − 1 mrad, requiring a deeper focal offset to lower the laser track below the surface. Overall, much higher conical phase front angles are required than available with the present beam delivery system (i.e. 20% loss at $\uptheta$ ~ ± 4 mrad; Fig. 4) to reach the full potential for filament lengths of > 100 μm for such shallow focussing.

For 1500 and 2500 µm focussing depth, the conical phase front induced similar trends of filament stretching on negative angles and condensing on positive angles (Fig. 5) as reported for the 600 µm deep focussing case (Fig. S2). In the 600 to 2500 µm depth range, filament tracks (Fig. 5) were either shortened (~ 50%) or lengthened (by up to 5$\times$) according to the respective positive or negative sign of the conical phase front angle. The longest and most uniform filaments are highlighted for each energy by the coloured ovals, showing a progression of increasing filament length from 121 µm with θ = − 5.59 mrad angle for 600 µm depth to 173 µm length at θ = − 0.83 mrad angle for 2500 µm depth. With deeper focussing, less conical phase front is required with the increasing stretching induced by the surface aberration. This scaling up of filament length was most rewarding for shallow focussing due to weakened surface aberration.

For positive angles, the conical phase fronts demonstrated an increasingly strong correction of surface aberration, on which Kerr and plasma focussing effects could be recovered even in the case of 2.5 mm deep focussing. In this way, filament lengths (Fig. 5) were shortened approximately by twofold from the case of non-modified beams ($\uptheta$ = 0 mrad) to lengths of ~ 11 and 59 µm for depths scaling from 600 to 2500 µm depth, respectively, and for angles scaling from + 3.94 to + 2.38 mrad, respectively. Shorter modification zones may not be possible even with other beam shapes as the presently high laser energy (8 μJ) experiences a stronger interplay of Kerr lensing and plasma defocussing with shrinking DOF.

The collective observation of track lengths in Fig. S2 and 5 points to two opposing aspects of processing wherein surface aberration and conical phase front can either play in concert to stretch the interaction volume (i.e. negative conical angle) or compensate each other’s elongation effects (i.e. positive conical angle). When negative conical angle was optimized for a particular focussing depth, a maximum length of uniform modification was demonstrated (coloured ovals, Fig. 5 and Fig. S2) without influence from nonlinear beam steering effects such as Kerr lensing and plasma defocussing. An optimum conical angle to enable this condition was demonstrated at each depth, but also was tuned to maximize the track length according to the available pulse energy. In this way, uniform modification tracks could be extended to any length, contained only by the ratio of the pulse energy and simulated track length (averaging 15 µm length per 1 µJ of pulse energy in this work). These affects will play out similarly for all focussed light beams, with trend lines varying more strongly with an increase in the NA of the focussing beam due to increased surface aberration.

### Tuning Kerr and plasma focussing

In assessing the morphology of laser tracks formed over the large range of conical phase front angles (i.e. $\uptheta$ = − 6.6 to + 5.6 mrad), focussing depths (up to z = 2500 µm depth) and pulse energies (~ 0.2 to 7.2 µJ), an energy threshold, E_cr_(θ,z), could be identified for the onset of nonlinear beam focussing effects due to Kerr and plasma for each angle and depth. These threshold energies encompass the inhibition or enhancement of nonlinear beam focussing as the peak focal intensity falls or rises by the combination of surface aberration and conical beam shaping effects.

The threshold energies (E_cr_(θ,z)) are represented on a false colour scale in a two-dimensional map (circles in Fig. 6) against the conical phase front angle and focussing depth. The black dashed line marks the onset threshold for surface ablation dominating over internal Kerr lensing. The minimum energy threshold rises from 270 nJ at 44 µm depth to 3.6 µJ energy at 2500 µm depth, following along a trend line of positive angle increasing from 0 to 2.83 mrad over the 0 to 2500 µm depth range, respectively. These angles closely align with the path of optimal conical angle, θ_opt_(z), (white dashed line in Fig. 6) found to best compensate for the surface aberration and thereby minimize the DOF, defining DOF_min_(θ_opt_(z), z).

The critical energy for Kerr lensing, E_cr_(θ,z), is expected to scale with depth and conical angle, rising over the baseline value^50^ of E_cr_(θ = 0, z = 0) = 221 nJ by the DOF elongation ratio according to E_cr_(θ,z) = E_cr_(0,0) · DOF(θ,z)/DOF(0,0). Following along the line for strongest focussing (white dashed in Fig. 6), the critical energy, E_cr_(θ,z) scaled up from ~ 221 nJ at 44 µm depth (θ = 0 mrad) to 850 nJ at 1500 µm depth (θ $\cong$ 1.4 mrad), which coarsely followed the observations (Fig. 6).

The calculation of focal elongation, DOF(θ,z), approximately follows a linear dependence on increasing depth and magnitude of conical angle, permitting a generalized representation of experimental date in Fig. 6 by two intersecting surfaces, as shown in false colour. Although there is a margin of error on establishing the onset for Kerr lensing, the threshold energy (coloured circles) demonstrates a global fit to the overall trend in which the onset energy for Kerr lensing must be scaled up with increasing focussing depth and magnitude of conical phase front angle. The onset energy must also account for aberration compensation by following the white dashed line in Fig. 6.

## Discussion

### Processing windows for Conical Phase Front

In this study, conical beam phase front was found to strongly interplay with surface aberration and nonlinear optical effects to favourably manipulate the ultrafast laser light propagation and dissipation, creating well-controlled and novel geometric morphologies in bulk fused silica glass. Surface aberration was purposely harnessed to elongate laser filament tracks as previously observed in optical fibre^51^. However, the uniformity and length of such tracks were significantly improved with negative conical phase fronts (Figs. 4, 5, and Fig. S2), while also inhibiting distortion effects from Kerr lensing and plasma defocussing (Figs. 3, 4, and Fig. S1).

A global summary of the tuning benefits of conical phase front are presented in Fig. 7 for generating long uniform filament tracks or minimizing the depth of focus for focal depths up to 2.5 mm. The optimal zone for generating long and uniform filaments (Fig. 7, vertical bars for 90% of maximum length) was provided by a reducing magnitude of angle in the concave conical phase front with decreasing depth. For 8 µJ pulse energy, the conical angle shifted ~ fivefold from − 1 $\pm$ 0.6 mrad for 2500 µm depth to − 5.6 mrad for 600 µm depth. The resulting laser tracks (Figs. 4 and 5) were longer and more uniform than possible by Kerr lensing or aberration alone. The conical beam stretching offered benefits up to the maximum focal depth of 2500 µm (Fig. 5), providing 1.4$\times$ elongation with 8 µJ pulse energy. The conical beam stretching was most effective for shallow focussing, where surface aberration effects were weakest, providing a 4.8× elongation for 600 µm depth and 8 µJ pulse energy. The benefits also improved with increasing pulse energy (Fig. 7) with optimal conical angle increasing from − 0.41 to − 5.6 mrad for pulse energy increasing from 1 to 8 µJ at 600 µm depth. Disabling of the Kerr and plasma focussing effects were key benefits of the conical phase front in avoiding broken filament tracks for shallow to modest focussing depth (i.e. < 600).

A further significant benefit of conical phase front tuning was the partial compensation of surface aberration, offering up to a twofold shortening of the laser interaction zone (Fig. 5) for 600 µm to 2500 µm depths. Figure 7 plots the compensation angle observed to produce the shortest laser track (Figs. 5, 6, Fig. S1) at the modification threshold energy. The optimal angle scaled from 0 $\pm$ 0.31 to + 1.76 $\pm$ 0.73 mrad with focal depth increasing from 200 µm to 2500 µm, respectively. As expected, the data align closely with the optimal conical phase front angle (blue line) for minimized DOF, DOF_min_(θ_opt_(z), z). The correction of surface aberration by the conical phase front will grow more challenging for deeper focussing depth (> 2.5 mm) owing to an increasing axial oscillation of the aberrated beam relative to shallower focussing (i.e., Fig. 3c at 600 µm depth).

The SLM presents a flexible alternative to the fixed axicon, enabling rapid and facile optimization of the conical phase angle to precisely meet the variable fabrication depth and pulse energy requirements when generating long filament tracks. SLM beam shaping thus enables a fuller harvesting of the available laser pulse energy for creating uniform and long filament tracks over shallow to deep focussing depth, unconstrained by the surface aberration. The results offer immediate benefits for boosting the quality, speed and overall process control in diverse applications such as cutting^18,40^, scribing^17^, photonics manufacturing, and micro- to nano-structuring of open channels^11^ over shallow to deep processing ranges.

The results further demonstrated that conical phase fronts are a flexible means for partial correction of surface aberration effect^22–24^ while also serving to manipulate Kerr lensing and plasma defocussing effects when higher pulse energies are available. Testing of other non-conical phase fronts shapes will be beneficial in learning how to further manipulate the complex interplay of light interactions that control the final focal volume shape in this complex domain of aberrated nonlinear beam propagation and dissipation. An all-encompassing beam shaping model for varying energy and focal depth is increasingly rewarding for controlling the larger interaction volumes now possible with the emergence today of kilowatt class ultrafast lasers^52^.

## Methods

### Experimental setup and beam shaping

The experimental arrangement for modifying the ultrafast laser beam with an SLM is shown in Fig. 1. Concave and convex conical phase fronts were applied to form single-pulse filaments in fused silica over a wide range of focal depths and pulse energies. The beam delivery system (Fig. 1a) was fed with an ytterbium-doped fibre laser (Amplitude Systems; Satsuma), providing a Gaussian intensity profile with beam quality factors of ${M}_{x}^{2}$ = 1.14 and ${M}_{y}^{2}$ = 1.01. The laser beam was frequency doubled to 515 nm wavelength to yield 250 fs pulses with maximum pulse energy of 10 µJ and variable repetition rate up to 2 MHz.

The output beam was expanded (not shown) to 10 mm spot size ($1/{e}^{2}$) to fill the 12 × 16 mm^2^ aperture (600 × 800 pixels of 20 µm size) of the liquid–crystal-on-silicon (LCOS) phase shifting SLM (Hamamatsu; X10468-04). A linear radial phase ramp generated a variable complementary conical angle, θ, with respect to a flat surface as defined in Fig. 1b,c, applied over ranges of 0 to + 6.6 mrad and 0 to − 6.6 mrad, respectively. The peak angles were limited by beam delivery apertures (mirrors and lenses) that reduced the relative transmission to 57% and 62%, respectively. A 4f-system of two lenses, *l*_1_ and *l*_2_, with focal lengths *f*_1_ = 85 cm and *f*_2_ = 35 cm, respectively, relayed the SLM-shaped beam (SLM plane) to the back focal plane of the aspherical fabrication lens *l*_3_ (*f*_3_ = 4.5 mm, 0.55 NA, Newport; 5722-A-H) with de-magnification of $\frac{{f}_{1}}{{f}_{2}}=\frac{85 cm}{35 cm}\approx 2.43$. The fabrication lens completed a final Fourier transform to generate the Vortex beam with the ring pattern as shown by the Fourier simulation in Fig. 1d when positioned at the focal plane (z = 0) inside of a fused silica substrate. The patterns were similar for the two opposite angles of $\uptheta =\pm 0.34\mathrm{ mrad}$. Fourier propagation simulation showed the angularly dispersed rays to converge after (Fig. 1b, left) or prior to (Fig. 1c, left) the focal plane position (white dashed line), generating a 0th order Gaussian–Bessel like beam. The peak intensity of Gaussian–Bessel beam was found to surpass the intensity of the Vortex beam except for the smallest angles (i.e. |$\uptheta$| < 0.17 mrad). In this way, laser interactions were dominated by the filament shape of the Gaussian-Bessel beam, while the length could be flexibly tuned simply by changing the conical angle, $\uptheta$.

Gaussian beam analysis without surface aberration predicted a DOF (full width at half maximum) of ~ 2.6 µm forming inside of fused silica. Fourier propagation of the Gaussian beam through the lens aperture was further applied to evaluate the intensity profile forming in the focal volume inside of the glass under the influence of both surface aberration and conical phase front. In the simulation, a surface aberration phase front for a given focussing depth^23^ was superimposed with the conical phase front (θ′ = 2.43 × θ) onto the incoming beam appearing at the first focal plane before (SLM image plane, Fig. 1a) the aspherical fabrication lens. Then, the Fourier propagation provide the intensity distribution in the vicinity of the second focal plane without considering optical nonlinear effects in the glass. Such simulation provided the focal elongation, DOF(θ,z), arising with increasing focal depth, z, and conical phase front angle$,\uptheta$. Transverse and axial beam profiles were recorded experimentally (Ophir; SP90422) and compared with simulated intensity profiles as presented in Fig. 1. After correcting for the de-magnified ratio, $\frac{{f}_{2}}{{f}_{3}}=\frac{350 mm}{4.5 mm}=77.78,$ and the refractive index ratio for glass to air, 1.46, the transverse (Fig. 1e) and longitudinal (Fig. 1b,c; right) beam profiles anticipated in shallow focussing (i.e. aberration free), near the equivalent Fourier plane inside of the glass target due to fabrication lens, *l*_3_, are presented for the respective θ = + 0.34 and − 0.34 mrad cases. The observed longitudinal profiles (Fig. 1b,c, right) match closely with the respective simulations for θ = + 0.34 (Fig. 1b, left) and − 0.34 (Fig. 1c, left) mrad, falling short in length by only ~ 18% and 10%, respectively.

### Fabrication and characterization

Laser interactions were studied in stacks of 1 mm thick fused silica (Precision Glass and Optics; $5.08 1.27 {\mathrm{cm}}^{2}$, Optical Grade) samples to support focussing depths up to 3 mm thickness. Refractive index matching oil was applied between samples and surfaces were aligned perpendicular to laser propagation direction to ± 0.2 mrad precision by monitoring the back reflected laser light from the glass surface with a second CCD camera (not shown in Fig. 1a). The positioning and translation of samples were precisely controlled by a 3-axis motion stage (Aerotech; PlanarDL-200 XY and ANT130-L-ZS).

For calibration of filament effects without conical phase front distortion, single pulses with flat wavefronts (θ = 0 mrad) were applied in the 0.27 to 3.04 µJ pulse energy range at finely spaced depths increasing in 14.6 µm increments from the top to bottom of 1 mm thick glass. Conical phase fronts were generated by the SLM, varying from θ = − 6.6 to + 6.6 mrad, forming filament-like intensity profiles that were applied over pulse energies of 0.5 to 8 µJ and focal depths of 200, 600, 1500, and 2500 μm. These exposures exceeded the critical pulse energy for self-focussing by up to 40×, calculated to be E_cr_ = 221 nJ for a nonlinear refractive index of ${n}_{2}=3\times {10}^{-16}$ cm^2^/W based on a similar 514 nm source^53^.

An optical microscope (Olympus; BX51) was used for studying the morphology and physical sizes of filaments.

## Supplementary Information

Supplementary Figures.

## References

[1] Booth MJ, Neil MAA, Wilson T (1998). Aberration correction for confocal imaging in refractive-index-mismatched media. J. Microsc..

[2] Rayner D, Naumov A, Corkum P (2005). Ultrashort pulse non-linear optical absorption in transparent media. Opt. Express.

[3] Li J, Ertorer E, Herman PR (2019). Ultrafast laser burst-train filamentation for non-contact scribing of optical glasses. Opt. Express.

[4] Herman, P. R., Marjoribanks, R. & Oettl, A. Burst-ultrafast laser machining method. U.S. Patent 6,552,301 (2003).

[5] Gattass RR, Mazur E (2008). Femtosecond laser micromachining in transparent materials. Nat. Photonics.

[6] Shimotsuma Y, Kazansky PG, Qiu J, Hirao K (2003). Self-organized nanogratings in glass irradiated by ultrashort light pulses. Phys. Rev. Lett..

[7] Taylor RS (2003). Femtosecond laser fabrication of nanostructures in silica glass. Opt. Lett..

[8] Taylor R, Hnatovsky C, Simova E (2008). Applications of femtosecond laser induced self-organized planar nanocracks inside fused silica glass. Laser Photon. Rev..

[9] Kumar K (2014). Quantized structuring of transparent films with femtosecond laser interference. Light Sci. Appl..

[10] Couairon A, Mysyrowicz A (2007). Femtosecond filamentation in transparent media. Phys. Rep..

[11] Bhuyan MK (2010). High aspect ratio nanochannel machining using single shot femtosecond Bessel beams. Appl. Phys. Lett..

[12] Sugioka K, Cheng Y (2014). Ultrafast lasers-reliable tools for advanced materials processing. Light Sci. Appl..

[13] Haque M, Lee KKC, Ho S, Fernandes LA, Herman PR (2014). Chemical-assisted femtosecond laser writing of lab-in-fibers. Lab Chip.

[14] Wu D (2015). In-channel integration of designable microoptical devices using flat scaffold-supported femtosecond-laser microfabrication for coupling-free optofluidic cell counting. Light Sci. Appl..

[15] Flamini F (2015). Thermally reconfigurable quantum photonic circuits at telecom wavelength by femtosecond laser micromachining. Light Sci. Appl..

[16] Chen F, de Aldana JRV (2014). Optical waveguides in crystalline dielectric materials produced by femtosecond-laser micromachining. Laser Photon. Rev..

[17] Hosseini, S. A. & Herman, P. R. Method of material processing by laser filamentation. U.S. Patent 9,296,066 (2016).

[18] Ahmed F, Lee MS, Sekita H, Sumiyoshi T, Kamata M (2008). Display glass cutting by femtosecond laser induced single shot periodic void array. Appl. Phys. A Mater. Sci. Process..

[19] Chung SH, Mazur E (2009). Surgical applications of femtosecond lasers. J. Biophotonics.

[20] Booth MJ (2014). Adaptive optical microscopy: The ongoing quest for a perfect image. Light Sci. Appl..

[21] Hnatovsky C (2005). High-resolution study of photoinduced modification in fused silica produced by a tightly focused femtosecond laser beam in the presence of aberrations. J. Appl. Phys..

[22] Chen YC (2017). Laser writing of coherent colour centres in diamond. Nat. Photonics.

[23] Huang L, Salter PS, Payne F, Booth MJ (2016). Aberration correction for direct laser written waveguides in a transverse geometry. Opt. Express.

[24] Cumming BP (2014). Adaptive optics enhanced direct laser writing of high refractive index gyroid photonic crystals in chalcogenide glass. Opt. Express.

[25] Osellame R (2003). Femtosecond writing of active optical waveguides with astigmatically shaped beams. J. Opt. Soc. Am. B.

[26] Ams M, Marshall G, Spence D, Withford M (2005). Slit beam shaping method for femtosecond laser direct-write fabrication of symmetric waveguides in bulk glasses. Opt. Express.

[27] Salter PS (2012). Adaptive slit beam shaping for direct laser written waveguides. Opt. Lett..

[28] Salter PS, Booth MJ (2012). Focussing over the edge: Adaptive subsurface laser fabrication up to the sample face. Opt. Express.

[29] Ren H, Lin H, Li X, Gu M (2014). Three-dimensional parallel recording with a Debye diffraction-limited and aberration-free volumetric multifocal array. Opt. Lett..

[30] Xu B (2016). High efficiency integration of three-dimensional functional microdevices inside a microfluidic chip by using femtosecond laser multifoci parallel microfabrication. Sci. Rep..

[31] Zhang C (2016). Optimized holographic femtosecond laser patterning method towards rapid integration of high-quality functional devices in microchannels. Sci. Rep..

[32] Mathis A (2012). Micromachining along a curve: Femtosecond laser micromachining of curved profiles in diamond and silicon using accelerating beams. Appl. Phys. Lett..

[33] Yamada K, Watanabe W, Toma T, Itoh K, Nishii J (2001). In situ observation of photoinduced refractive-index changes in filaments formed in glasses by femtosecond laser pulses. Opt. Lett..

[34] Kumagai M (2007). Advanced dicing technology for semiconductor wafer—Stealth dicing. IEEE Trans. Semicond. Manuf..

[35] Tamaki T, Watanabe W, Nishii J, Itoh K (2005). Welding of transparent materials using femtosecond laser pulses. Japan. J. Appl. Phys. Part 2 Lett..

[36] Duocastella M, Arnold CB (2012). Bessel and annular beams for materials processing. Laser Photon. Rev..

[37] He F (2017). Tailoring femtosecond 1.5-μm Bessel beams for manufacturing high-aspect-ratio through-silicon vias. Sci. Rep..

[38] Yao Z (2018). Non-diffraction-length, tunable, Bessel-like beams generation by spatially shaping a femtosecond laser beam for high-aspect-ratio micro-hole drilling. Opt. Express.

[39] Dudutis J, GeČys P, RaČiukaitis G (2016). Non-ideal axicon-generated Bessel beam application for intra-volume glass modification. Opt. Express.

[40] Mishchik K (2017). Improved laser glass cutting by spatio-temporal control of energy deposition using bursts of femtosecond pulses. Opt. Express.

[41] Liu X (2018). Front-surface fabrication of moderate aspect ratio micro-channels in fused silica by single picosecond Gaussian-Bessel laser pulse. Appl. Phys. A Mater. Sci. Process..

[42] Ashcom JB, Gattass RR, Schaffer CB, Mazur E (2006). Numerical aperture dependence of damage and supercontinuum generation from femtosecond laser pulses in bulk fused silica. J. Opt. Soc. Am. B.

[43] Lapointe J, Kashyap R (2017). A simple technique to overcome self-focusing, filamentation, supercontinuum generation, aberrations, depth dependence and waveguide interface roughness using fs laser processing. Sci. Rep..

[44] Kammel R (2014). Enhancing precision in fs-laser material processing by simultaneous spatial and temporal focusing. Light Sci. Appl..

[45] Vitek DN (2010). Spatio-temporally focused femtosecond laser pulses for nonreciprocal writing in optically transparent materials. Opt. Express.

[46] Salter PS, Booth MJ (2012). Dynamic control of directional asymmetry observed in ultrafast laser direct writing. Appl. Phys. Lett..

[47] Patel A, Svirko Y, Durfee C, Kazansky PG (2017). Direct writing with tilted-front femtosecond pulses. Sci. Rep..

[48] Hnatovsky C (2005). Pulse duration dependence of femtosecond-laser-fabricated nanogratings in fused silica. Appl. Phys. Lett..

[49] Couairon A, Sudrie L, Franco M, Prade B, Mysyrowicz A (2005). Filamentation and damage in fused silica induced by tightly focused femtosecond laser pulses. Phys. Rev. B.

[50] Yariv A (1988). Quantum Electronics.

[51] Ertorer E, Haque M, Li J, Herman PR (2018). Femtosecond laser filaments for rapid and flexible writing of fiber Bragg grating. Opt. Express.

[52] Müller M (2016). 1 kW 1 mJ eight-channel ultrafast fiber laser. Opt. Lett..

[53] Milam D (1998). Review and assessment of measured values of the nonlinear refractive-index coefficient of fused silica. Appl. Opt..

