# Supplementary material for: Inhibition and enhancement of linear and nonlinear optical effects by conical phase front shaping for femtosecond laser material processing
Source: Sci Rep. 2020 Dec 9;10:21528. doi: 10.1038/s41598-020-78373-4 (PMC7726100; doi:10.1038/s41598-020-78373-4)
Supplement: Supplementary file 1 — Supplementary Figures. [file 41598_2020_78373_MOESM1_ESM.pdf]

Title: Inhibition and Enhancement of Linear and Nonlinear Optical Effects by Conical Phase Front Shaping for Femtosecond Laser Material Processing

Authors: Ehsan Alimohammadian*, Erden Ertorer, Erick Mejia Uzeda, Jianzhao Li, Peter R. Herman

Authors affiliation:

Department of Electrical and Computer Engineering, University of Toronto

10 King's College Road, Toronto, Ontario, Canada M5S 3G4

Supplementary 1

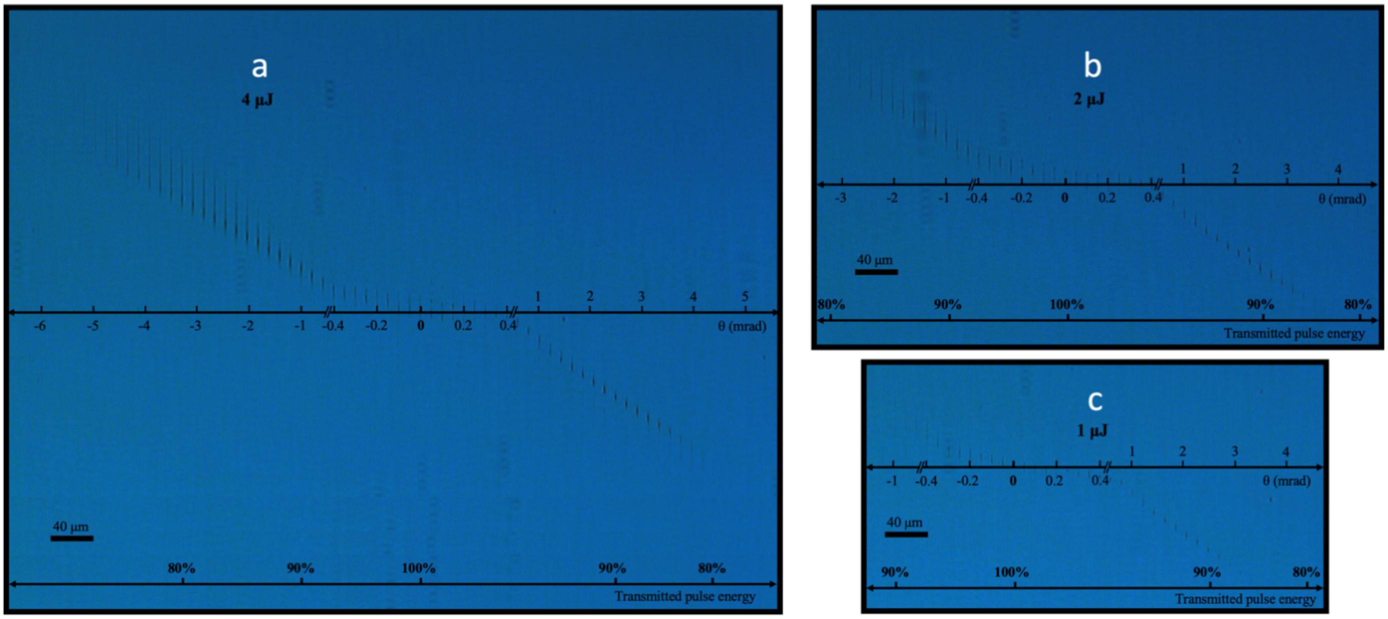

Figure S1. Laser modification tracks in glass manipulated by conical phase front angle and pulse energy. Sequence of modification tracks by backlighting optical microscopy, generated in fused silica by single laser pulses of increasing pulse energy, 4µJ (a), 2 µJ (b), and 1 µJ (c), at 600 µm focal depth, plotted with varying conical phase front angle from −6.6 to +5.6 mrad. Comparing with Fig. 3 for 8 µJ case, higher pulse energy is seen to favour selection of steeper concave phase fronts to generate the longest and most uniform filament tracks. The horizontal axes mark the depth of peak intensity under surface aberration for the unmodified beam (θ = 0). The bottom horizontal axes mark the optical system transmittance, reduced by conical beam divergence through apertures in the 6f system.

Supplementary 2

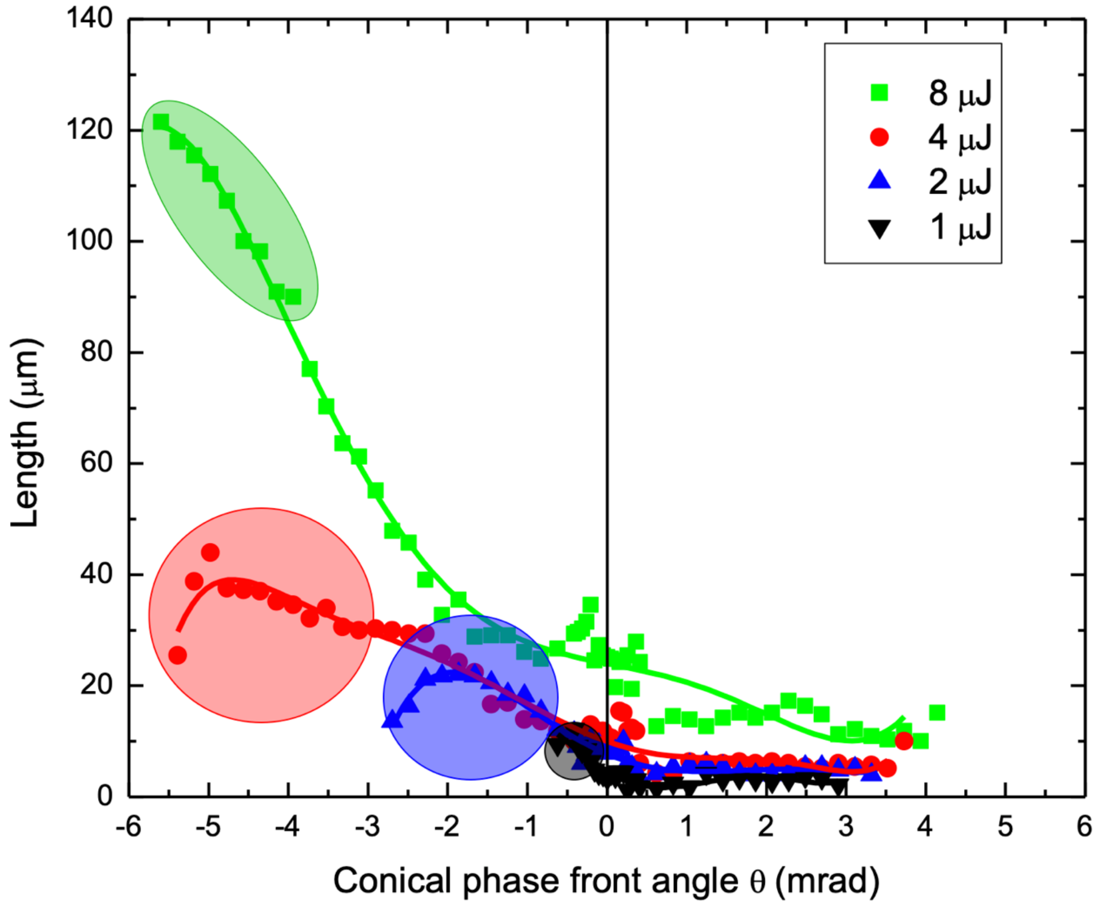

Figure S2. Influence of conical phase front on single-pulse filament length for varying laser pulse energy. Length of filament tracks formed by single pulses of 8 μJ (green), 4 μJ (red), 2 μJ (blue), and 1 μJ (black) energy observed under varying conical phase front angle, θ, presented for 600 μm depth. The optimal zones for long and uniform filament tracks (oval or circle sections) shift to an increasingly negative angle with increasing pulse energy. Solid lines are a guide for the eye.
